# High-Throughput In Vitro Screening of Changed Algal Community Structure Using the PhotoBiobox

**DOI:** 10.4014/jmb.2006.06027

**Published:** 2020-08-21

**Authors:** Dae-Hyun Cho, Kichul Cho, Jina Heo, Urim Kim, Yong Jae Lee, Dong-Yun Choi, Chan Yoo, Hee-Sik Kim, Seunghee Bae

**Affiliations:** 1 Cell Factory Research Center, Korea Research Institute of Bioscience & Biotechnology (KRIBB), Daejeon 344, Republic of Korea; 2 Department of Environmental Biotechnology, KRIBB School of Biotechnology, Korea University of Science and Technology (UST), Daejeon 34113, Republic of Korea; 3 Department of Genetic Resources Research, National Marine Biodiversity Institute of Korea, Seocheon-gun, Republic of Korea; 4 Department of Chemical and Biomolecular Engineering, KAIST, Daejeon 305-701, Republic of Korea; 5 Research Institute for Molecular-Targeted Drugs, Department of Cosmetics Engineering, Konkuk University, Seoul 0029, Republic of Korea

**Keywords:** PhotoBiobox, temperature, light, microalgae, polyculture, cultivation

## Abstract

In a previous study, the sequential optimization and regulation of environmental parameters using the PhotoBiobox were demonstrated with high-throughput screening tests. In this study, we estimated changes in the biovolume-based composition of a polyculture built in vitro and composed of three algal strains: *Chlorella* sp., *Scenedesmus* sp., and *Parachlorella* sp. We performed this work using the PhotoBiobox under different temperatures (10-36°C) and light intensities (50-700 μmol/m^-2^/s^-1^) in air and in 5% CO_2_. In 5% CO_2_, *Chlorella* sp. exhibited better adaptation to high temperatures than in air conditions. Pearson’s correlation analysis showed that the composition of *Parachlorella* sp. was highly related to temperature whereas *Chlorella* sp. and *Scenedesmus* sp. showed negative correlations in both air and 5% CO_2_. Furthermore, light intensity slightly affected the composition of *Scenedesmus* sp., whereas no significant effect was observed in other species. Based on these results, it is speculated that temperature is an important factor in influencing changes in algal polyculture community structure (PCS). These results further confirm that the PhotoBiobox is a convenient and available tool for performance of lab-scale experiments on PCS changes. The application of the PhotoBiobox in PCS studies will provide new insight into polyculture-based ecology.

## Introduction

Over the last few decades, microalgal biomass has attracted much attention as an eco-friendly and sustainable resource for use in biofuels, cosmetics, nutraceuticals, biofertilizers and aquaculture feed due to distinguishing advantages that include a fast growth rate, efficient removal of atmospheric CO_2_, and the accumulation of many valuable compounds [1, 2]. Among these, the concept of biofuel production using microalgae is regarded as a promising future technology as the cultivation of microalgae does not require arable lands or compete with many food resources [3]. Furthermore, they accumulate more lipid content than most terrestrial plants, which can be converted to biodiesel via transesterification [3]. However, microalgae-based industrial applications are still in their infancy because low biomass productivity and low target metabolite accumulation limit their use. To overcome this problem, many studies have focused on enhancing algal biomass and metabolite productivity. These studies encompass genetic engineering of selected microalgae, stress induction, the construction of new cultivation apparatus such as the photobioreactor, and the hybrid operation of raceway open pond systems [2,4-6]. Furthermore, the polyculture of varying microalgal species in the same cultivation apparatus has also been conducted to cope with the limitations of established mono-algal culture systems. According to Novoveská *et al*., mixing two or more algal strains in a polyculture system effectively increases and stabilizes algal biomass productivity compared to that of mono-algal cultures [7]. Moreover, mixed cultures of *Chlorella* sp. with the yeast *Saccharomyces cerevisiae* showed a higher specific growth rate than mono-algal cultures with higher oil productivity under 1,000 lux of red LED light [8]. Thus, if strains do not inhibit each other, this concept can be effectively applied to outdoor algal cultivation and wastewater treatment systems, which are subject to variable environmental conditions, as each strain exhibits different adaptations to changes in temperature, light intensity, and other environmental factors. To optimize the biomass productivity of polyculture systems, lab-scale testing to identify the dominant species under different environmental conditions supports the monitoring of algal polyculture fluctuations and biomass productivity. However, monitoring polycultures in lab-scale experiments is highly difficult owing to a lack of experimental apparatus such as incubators.

In a previous study, we designed the PhotoBiobox equipped with an LED array and light gradient filter, along with temperature and gas flow regulators to screen the optimal culture conditions of isolated microalgae using a 96-well microplate [9]. The study demonstrated that the PhotoBiobox can easily, automatically, and simultaneously regulate light intensity, temperature, and gas supply level to help determine the optimal culture conditions of target microalgae. Based on that study, we hypothesized that the PhotoBiobox can also be applied as an in vitro tool to study the monitoring of fluctuations in algal polyculture systems. Thus, in a further study, we investigated the changes in polyculture community structure (PCS) under simultaneous and sequential fluctuations of temperature and light intensity, as well as different levels of CO_2_, using the PhotoBiobox. The investigated algal community was composed of three freshwater microalgae (*Parachlorella* sp., *Chlorella* sp., and *Scenedesmus* sp.) which had been considered to be dominant species in the outdoor open wastewater pond algal cultivation system used in the previous study [10]. To verify which environmental factors affect algal succession in the open pond mass cultivation system of the modified algal polyculture, different light intensities, temperatures, and CO_2_ concentrations were evaluated in this study. This newly developed approach will facilitate high-throughput analysis of dominant microalgal species and biomass productivity in engineered polyculture systems under various conditions, and provide essential information for community control in outdoor mass cultivation.

## Materials and Methods

### Algal Source and Cultivation

**Fig. 1:**
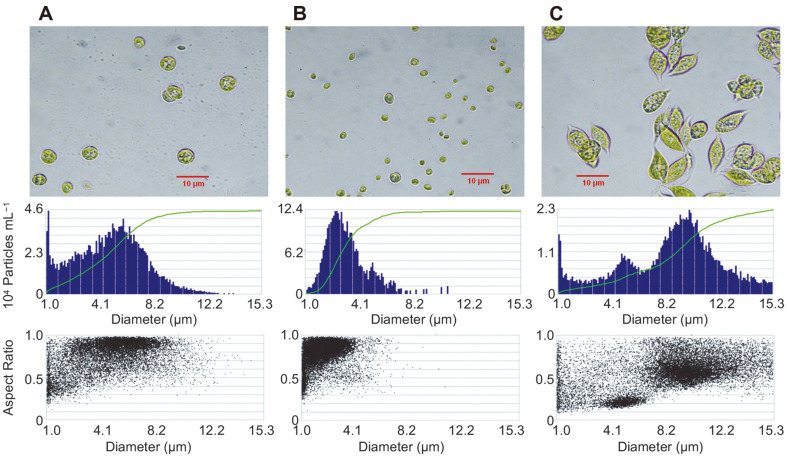
Morphological images, histograms and scatter plots of each algal strain, obtained by FlowCAM and based on three parameters: diameter (μm, X-axes), particles per ml (Y-axis of histograms), and aspect ratio (Y-axis of scatter plots); (A) *Parachlorella* sp., (B) *Chlorella* sp., and (C) *Scenedesmus* sp.

Strains including *Parachlorella* sp. JD-076 (KP091739) and *Scenedesmus* sp. YC001 (KC439160) were previously isolated from swine wastewater, and *Chlorella* sp. HS-2 (KU674363) was isolated from Jeju Island (Figs. 1A-1C) [9, 11]. Each strain was aseptically cultivated using a gamma-sterilized T25 Cell Culture Flask (SPL Life Sciences, Korea) with 20 ml of sterilized BG-11 medium and stored in a plant growth chamber (Jeio Tech, Korea) regulated at 26°C under 50 μmol/m^-2^/s^-1^ of continuous cool-white fluorescent light [12]. We used algal cells in the exponential growth phase for further PhotoBiobox experiments.

### Use of the PhotoBiobox to Analyze Algal Polyculture Changes

The PhotoBiobox can be easily controlled using the equipped touch-pad to regulate the temperature and light intensity and adjust the inlet gas-flow (Supplementary data 1). The temperature and light intensity were sequentially regulated ranging from 19.0°C to 36.0°C and from 50 μmol/m^-2^/s^-1^ to 700 μmol/m^-2^/s^-1^, respectively. The prepared 96-well microplates were incubated in the PhotoBiobox in air or 5% CO_2_ conditions, and the absorbance (680 nm) of each microalga was determined using a Synergy 2 microplate reader (BioTek, USA) after three days of incubation. To protect the culture medium against evaporation, the 96-well plate was covered with a transparent Breathe-Easy sealing membrane (Diversified Biotech, USA). The number of algal cells was estimated using the Bench Top FlowCAM (Fluid Imaging Technologies, Inc., USA). In order to determine the algal cell concentration, the equipment was manually primed with a 10-fold diluted sample, and the flow rate was adjusted at 0.5 ml/min^-1^. The detected cell concentration was verified by visible cell counting using a haemocytometer (Marienfeld, Germany) and the Eclipse 80i Optical Microscope (Nikon, Japan).

### Determination of Algal Biovolume

The biovolume of microalgae was estimated using the method proposed by previous studies [13, 14]. Microscopic observation was performed using an Eclipse T*i*-E inverted microscope (Nikon) and the length, width, and height of the microalgae determined from the microscopic images and Bench Top B3 series FlowCAM (Fluid Imaging Technologies, Inc.) data. The biovolume of each microalga was calculated according to shape-based equations [13]. Equal biovolumes of the three microalgal species were then mixed and inoculated into 96-well microplates. For the comparison with mono-culture condition, the same biovolume of each single microalga was also inoculated into 96-well microplates. Subsequently, the 96-well microplates were incubated in the PhotoBiobox.

### Statistical Analysis

High-throughput in vitro testing was performed in triplicate. Intraclass correlation coefficients (ICC) were calculated based on the Cronbach’s alpha and ICC model using SPSS version 18.0 for Figs. 3 and 4 as shown in supplementary data 5, 6 and 7. (α ≥ 0.9, excellent; 0.9 > α ≥ 0.8, good; 0.8 > α ≥ 0.7, acceptable; 0.7 > α ≥ 0.6, questionable; 0.6 > α ≥ 0.5, poor; 0.5 > α, unacceptable). Pearson’s correlation analysis was performed using SPSS 18.0 software (SPSS Inc., USA) to determine the relationship between PCS composition and different environmental conditions (temperature and light intensity). We considered correlation values from 0.8 to 1.0 to indicate a very strong correlation, from 0.6 to 0.79 a strong correlation, from 0.4 to 0.59 a moderate correlation, from 0.2 to 0.39 a weak correlation, and from 0.0 to 0.19 a very weak correlation.

## Results

### Morphological Separation and Characterization of Algal Strains

Over the past decades, studies in biovolume-based analysis of algal community structure have been performed to predict the physicochemical factors affecting community changes [14-16]. According to Olenina *et al*., the inadequacy of using the cell concentration of phytoplankton has been recognized, and calculation of the biovolume of each species in a phytoplankton community is important to estimate phytoplankton biomass in routine monitoring of study areas [17]. Our previous studies on open pond-based microalgal cultivation using untreated municipal wastewater in high-rate algal pond systems also proved the use of biovolume as a measure of algal mass and dominance [10, 14]. During the cultivation periods, the diversity analysis of algal communities revealed that the dominant algal species were *Scenedesmus* sp., *Chlorella* sp., and *Parachlorella* sp. [10, 14]. Thus, we estimated changes to the biovolume-based algal community composition of an artificially made algal PCS composed of *Scenedesmus* sp., *Chlorella* sp., and *Parachlorella* sp. under PhotoBiobox-regulated conditions. As shown in Figs. 1A-1C, different sizes of algal cells were tested to predict PCS changes under different conditions. The size of each microalga was determined by a FlowCAM and verified by optical microscope. As shown in Figs. 1A-1C, *Parachlorella* sp., *Chlorella* sp., and *Scenedesmus* sp. were shown to have diameters of about 4.0-7.5 μm, 2.0-3.5 μm, and 4.0-12.5 μm, respectively. Two of the algal strains, *Chlorella* sp. and *Parachlorella* sp., exhibited a spherical shape (aspect ratio: above 0.7), whereas the *Scenedesmus* sp. was a prolate spheroid shape (aspect ratio: 0.3-0.7) (Figs. 1A-1C). Based on the aspect ratio and diameter of each microalga, scatter plot data and cell images were divided as shown in Supplementary data 2A-F. The *Parachlorella* sp. exhibited a relatively larger diameter than the *Chlorella* sp., whereas a similar aspect ratio was detected by the FlowCAM (Supplementary data 2B and 2C). The *Scenedesmus* sp. was separated into two types; 1) approximately 4.0-7.0 μm in diameter with an aspect ratio ranging from 0.1 to 0.3, and 2) approximately 7.0-12.5 μm in diameter with an aspect ratio ranging from 0.4 to 0.7 (Supplementary data 2D and 2E). Cell debris was detected with aspect ratios in the range of 0.25 to 0.7 and 0 to 6 μm diameter (Supplementary data 2F). The average biovolume of the microalgae was calculated based on each shape, and the biovolumes of *Chlorella* sp., *Scenedesmus* sp., and *Parachlorella* sp. were 65.5, 564.4, and 381.7 μm^3^, respectively. Because of the different biovolumes of the microalgae, the cell concentrations (cells/ml^-1^) of *Chlorella* sp., *Scenedesmus* sp., and *Parachlorella* sp. were 1.95 × 10^7^ cells/ml^-1^, 4.38 × 10^6^ cells/ml^-1^, and 9.80 × 10^6^ cells/ml^-1^ at the same absorbance value (0.2).

### Change in Microalgal Cell Density Via Carbon Dioxide Condition Under Various Light Intensity and Temperature Conditions

**Fig. 2:**
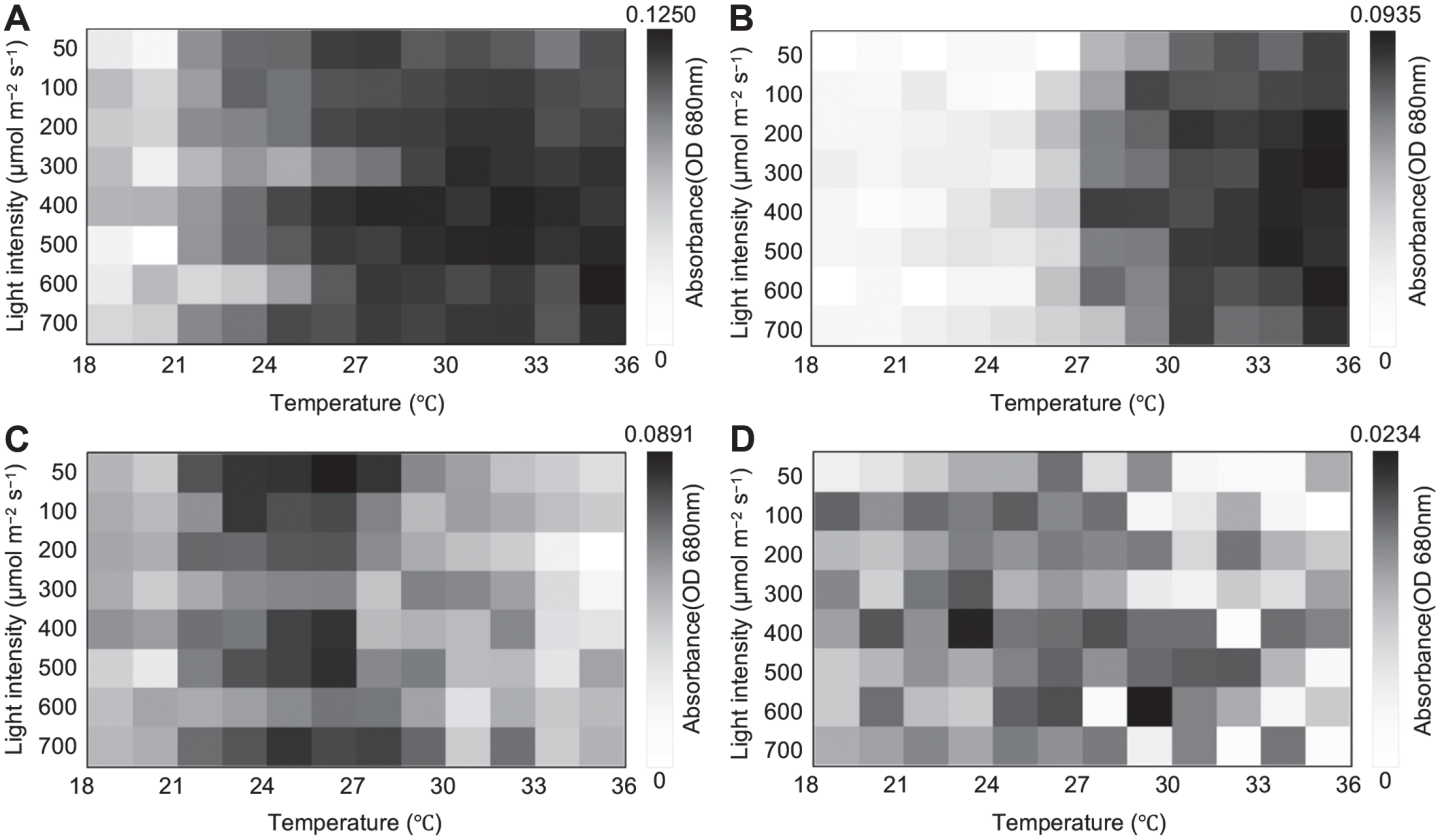
Heat map images of absorbance values; (A) mixed culture, (B) *Parachlorella* sp., (C) *Chlorella* sp., and (D) *Scenedesmus* sp. under different temperatures and light intensities in the air-flow condition. Experiment was performed in triplicate (*n* = 3).

**Fig. 3:**
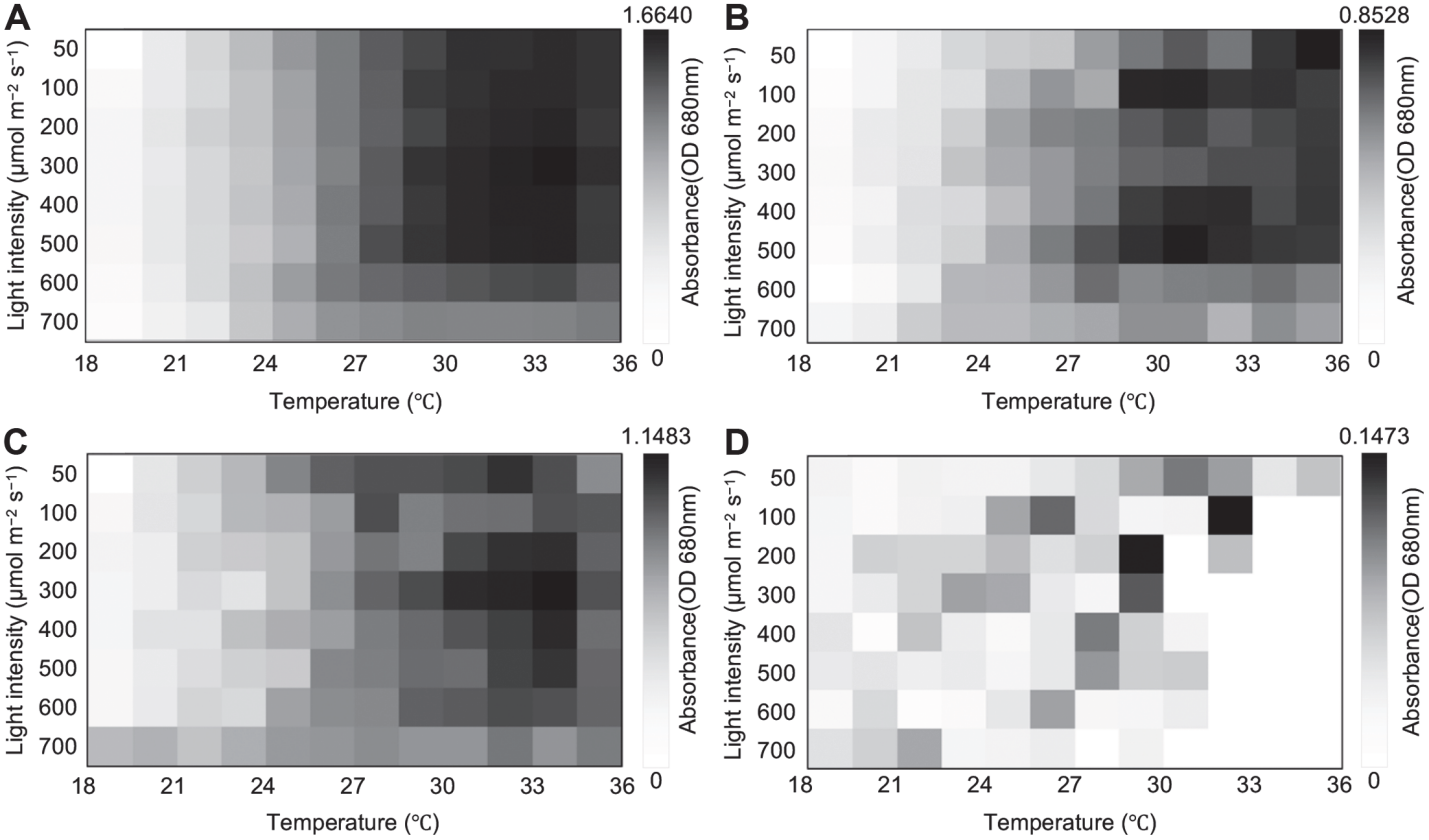
Heat map images of absorbance values; (A) polyculture (*Parachlorella* sp., *Chlorella* sp., and *Scenedesmus* sp.), (B) *Parachlorella* sp., (C) *Chlorella* sp., and (D) *Scenedesmus* sp. under different temperatures and light intensities in the 5% CO_2_ condition. Experiment was performed in triplicate (*n* = 3).

The changes in the daily cell density (absorbance values) are exhibited in Supplementary data 3 and 4. As similar absorbance values were exhibited after two days, the data after two days could be represented as heat map images divided by species, as shown in Figs. 2 and 3. Under air-flow conditions, the mixed culture showed relatively high absorbance at high temperatures (from 25.2°C to 36.0°C), and the optimal light intensity ranged from 300 μmol/m^-2^/s^-1^ to 600 μmol/m^-2^/s^-1^, indicating a slightly increased PCS size (Fig. 2A). Whereas *Parachlorella* sp. exhibited high absorbance values at high temperatures (28.3°C to 36°C), *Chlorella* sp. and *Scenedesmus* sp. showed high absorbance values at relatively lower temperatures (Figs. 2B-2D). Similarly, both the mixed culture and the *Parachlorella* sp. culture showed higher absorbance values at higher temperatures under 5% CO_2_ conditions (Fig. 3A). However, the absorbance of *Chlorella* sp. increased at higher temperatures under 5% CO_2_ conditions than it did under air-flow conditions, and the *Scenedesmus* sp. culture exhibited higher absorbance values at relatively lower temperatures under all aeration conditions (Figs. 3B-3D). Interestingly, the concentrations of atmospheric CO_2_ considerably affected the adaptation of *Chlorella* sp. against high temperatures, and photo-inhibition at a high light intensity (700 μmol/m^-2^/s^-1^) was apparent only under 5% CO_2_ conditions.

### Change in Polyculture Composition Structure (PCS) via Carbon Dioxide Condition Under Various Light Intensity and Temperature Conditions

**Fig. 4:**
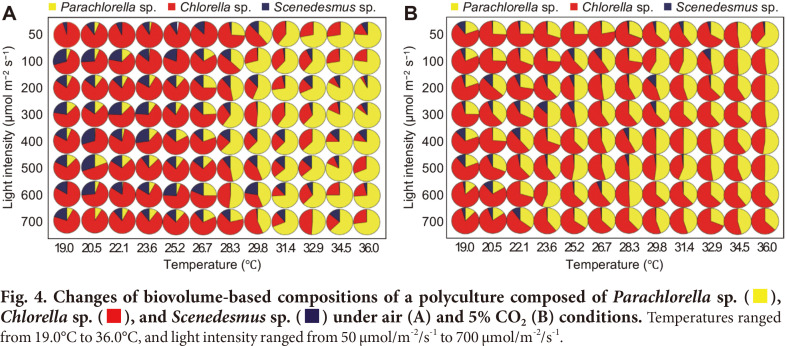


Figs. 4A and 4B present the simultaneous effect of temperature and light intensity on the compositional values of PCS. As shown Fig. 3A, the biovolume-based compositional values of *Parachlorella* sp. were relatively higher than other species at high temperatures, and *Chlorella* sp. showed high compositional values at lower temperatures. However, *Parachlorella* sp. and *Chlorella* sp. showed similar compositional values at high temperatures with a 5% CO_2_ supply (Fig. 4B). In the air-flow condition, *Parachlorella* sp. showed higher compositional values at high temperatures (26.7°C to 36°C) compared to other species, and no significant compositional changes were observed under different light intensities (Fig. 4A). As described above, in 5% CO_2_, *Chlorella* sp. showed relatively lower compositional values at high temperatures despite increasing cell growth, and the light intensity did not significantly affect compositional changes (Fig. 4B). The compositional values of *Scenedesmus* sp. were relatively lower than other species in both air and 5% CO_2_, but it did show slightly higher growth at lower temperatures. In Supplementary data 8, we presented each effect of temperature and light intensity on the compositional values. The compositional values of PCS present highly altered effects via change in temperature. However, it confirmed no or only slight effects via light intensity.

**Fig. 5:**
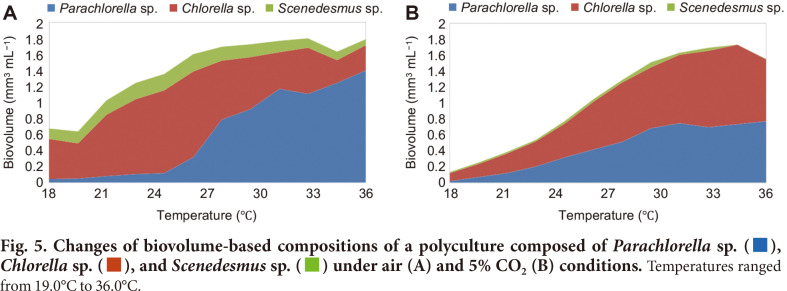


The Pearson’s correlation analysis of compositional values revealed that temperature showed a very strong positive correlation with the biovolume-based composition of *Parachlorella* sp. whereas negative correlations were found with *Chlorella* sp. and *Scenedesmus* sp., in both air and 5% CO_2_ (Table 1). As shown in Fig. 5, *Parachlorella* sp. was the dominant species at high temperatures under air-flow, whereas *Chlorella* sp. was the dominant species under 5% CO_2_. These results indicate that algal community structure can be significantly affected by the CO_2_ supply into the algal polyculture.

Light intensity showed a moderate positive correlation with *Scenedesmus* sp., whereas *Parachlorella* sp. and *Chlorella* sp. showed very weak correlations with air-flow conditions. However, in 5% CO_2_, *Scenedesmus* sp. showed a strongly negative correlation with light intensity, whereas other species did not show significant correlations (Table 1). The results revealed that compositional values of all three species were significantly affected by temperature, whereas light intensity only affected *Scenedesmus* sp. in the studied algal community.

## Discussion

A previous study reported that the biomass and chlorophyll *a*-based growth curve of *Chlorella vulgaris* ARC1 showed relatively high growth at 6% CO_2_, and it also showed tolerance to high temperatures (40 and 50°C) in 6%CO_2_, whereas no growth was observed at 50°C in 0.036% CO_2_ [18]. The study also reported increased photosynthetic pigment in 6% CO_2_, including chlorophyll a and carotenoid content, indicating increased photon capture in the algal cells [18]. It was also reported that the augmented water temperature generally increased the metabolic activity and growth of microalgae, but CO_2_ solubility decreases at high temperatures with lower values of pKa1 and pKa2 carbonic acid [19]. Based on these reports, we speculated that the different solubility of atmospheric CO_2_ and the changed metabolism of algal cells at different temperatures also lead to different patterns of algal adaptation in each algal culture (Figs. 3A-3D). Therefore, the changes in *Chlorella* sp. composition under 5% CO_2_ at high temperatures may be due to relatively high photosynthetic activity compared to that of other species. The relative selectivity factor (S_rel_), representing the capacity of ribulose bisphosphate carboxylase-oxygenase (RUBISCO) to discriminate between CO_2_ and O_2_, decreased at higher CO_2_ levels. RUBISCO is considered as a primary CO_2_-fixing enzyme, and it generates phosphoglycolate through oxygenase activity. The phosphoglycolate inhibits RUBISCO carboxylase activity and is alleviated by dephosphorylation via phosphoglycolate phosphatase. Thereafter, the generated glycolate is used for cellular metabolism via photorespiration or excreted from the algal cells [19-21]. The values of S_rel_ are generally dissimilar in different taxa because various species have evolved during different atmospheric CO_2_ levels over geological time [19, 22, 23]. Based on the previous reports, we consider the different algal growth patterns under air and 5% CO_2_ conditions in this study to be closely related to the different RUBISCO capacities of each microalga. This would affect their growth responses at different temperatures and light intensities in air or 5% CO_2_.

Comprehensive studies on photo-inhibition effects in microalgae have been performed in various laboratory conditions, and interestingly, in this study more photo-inhibition effects were apparent at a high light intensity (700 μmol/m^-2^/s^-1^) under 5% CO_2_ conditions (Fig. 3). Photo-inhibition generally occurs due to inhibition of photosystem II activity under high light intensities because of an imbalance between photo-damage and its repair system by generated reactive oxygen species (ROS) [24]. Previous studies showed that high light intensity coupled with a high CO_2_ concentration significantly increased cellular ROS generation in *Nannochloropsis salina*, along with elevated antioxidant compounds, when compared to those under low light intensities [25]. Although *N. salina* was not sufficiently damaged by ROS production, the study showed that increased oxidative stress can occur under the simultaneous effects of high light intensity and high CO_2_ concentration. Increased oxidative stress under high CO_2_ conditions is demonstrated by the acidification of the culture medium, and this event reduces the bicarbonate uptake of microalgae because the photosynthetic rate is highly affected by species-specific pH ranges [26]. A previous study also reported that a decrease in pH affects the activity of the xanthophyll cycle, and this event may have evolved in response to a high light-induced photo-inhibition effect [27]. The different biovolume-based compositional changes under air and 5% CO_2_ conditions might be closely related to the explained mechanisms. Because the optimal temperature of algal species changes under 5% CO_2_ conditions, it might affect compositional values at high temperatures and high light intensities. Based on the correlation analysis, we speculated that the enhanced growth of *Chlorella* sp. at elevated temperatures under 5% CO_2_ conditions might decrease the correlation values of *Parachlorella* sp. and *Scenedesmus* sp. (Table 1).

In the present study, we applied the PhotoBiobox in the study of algal PCS dynamics using lab-scale experiments. The PhotoBiobox is a tablet-sized and 96-well-based, high-throughput photobioreactor that was developed to screen for optimal high growth conditions by analyzing the algal biomass, CO_2_ sequestration and their metabolites including lipids in live growth conditions [9]. The advantages of the PhotoBiobox are that the optimal culture conditions including light intensity, temperature and CO_2_ level for the alga or algal community of interest can be derived without extra, time-consuming steps including sampling and analysis setting that are generally required for flow cytometric analysis (FlowCAM method). The conventional lab-scale method using a plant chamber and flasks is highly time-consuming and arduous because the experiment requires many apparatuses to test the enormous number of experimental groups. Also, limited information on simultaneous environmental effects has been reported from lab-scale experiments because this is hard to realize in an ordinary laboratory. Thus, PhotoBiobox-based lab-scale experimentation on algal PCS will provide new insights in microalgal polyculture-based biomass production and wastewater treatment, along with ecological and physiological studies.

In this study, we confirmed that *Chlorella* sp. and *Scenedesmus* sp. dominated at low temperatures and *Parachlorella* sp. dominated at high temperatures, indicating that common cultivation condition against each strain would be limited to maximized biomass productivity in the polyculture. Previous studies, as well as our own studies, also found that it was difficult to maintain optimal biomass productivity by a single species in a domestic environment with daily and yearly temperature difference [10, 14]. For these reasons, polyculture would be an efficient strategy for maintaining optimal productivity at wide temperatures while also benefiting microalgal biomass productivity. In outdoor culture, however, microalgae growth and biomass productivity are affected by various environmental factors (nutrients, grazers) in addition to temperature and light. Therefore, further studies are required to clarify the effects on different biomass productivities on single culture and polyculture systems.

## Conclusion

In this study, the changes in PCS under different temperatures and light intensities in air or CO_2_ conditions were investigated using the PhotoBiobox. The results revealed that atmospheric CO_2_ concentration can affect the environmental adaptation of microalgae. During several decades, many studies on temperature and light intensities have been performed in lab-scale conditions. However, these studies have provided only limited information on simultaneous effects and sequential community changes due to the limitations of the apparatus used. The PhotoBiobox enables easy application in a wide range of experiments as it is equipped with ample features including different LED light colors (blue, green, and red), simultaneous regulation of temperature and light intensity, self-mixing (vibration), gas-flow control, and a convenient touch-pad-based regulation system. Similar to our own study, future PhotoBiobox-based projects related to dynamic PCS will deepen our comprehensive understanding of algal mass cultivation. Physiological and ecological studies as well as biological studies of atmospheric CO_2_-derived climate change can be performed conveniently in lab-scale conditions with PhotoBiobox. In addition, further studies using the PhotoBiobox and regional environmental samples are required to examine the dynamics of harmful bloom-forming algae in lakes, rivers, and oceans for better understanding of phytoplankton community dynamics under specific environmental changes.

## Supplemental Materials



Supplementary data for this paper are available on-line only at http://jmb.or.kr.

## Figures and Tables

**Table 1:** Pearson’s correlations between the biovolume composition of microalgae strains and environmental parameters (temperature and light intensity) under air or 5% CO_2_ flow conditions (*n* = 12).

Strains	Air		5% CO_2_	

	Temperature	Light intensity	Temperature	Light intensity
*Parachlorella* sp.	0.951^**^	0.028	0.855^**^	0.068
*Chlorella* sp.	-0.931^**^	-0.024	-0.783^**^	0.105
*Scenedesmus* sp.	-0.969^**^	0.511	-0.948^**^	-0.696

^**^p < 0.01
