# Supplementary material for: High-Throughput In Vitro Screening of Changed Algal Community Structure Using the PhotoBiobox
Source: J Microbiol Biotechnol. 2020 Aug 21;30(11):1785–91. doi: 10.4014/jmb.2006.06027 (PMC9728362; doi:10.4014/jmb.2006.06027)

J. Microbiol. Biotechnol.
https://doi.org/10.4014/jmb.2006.06027

Supplementary data 1. PhotoBiobox equipped with temperature and light intensity or colour (white, blue, red, and green) regulators, gas supply inlet connections, and a touch pad for convenient experimental set up.

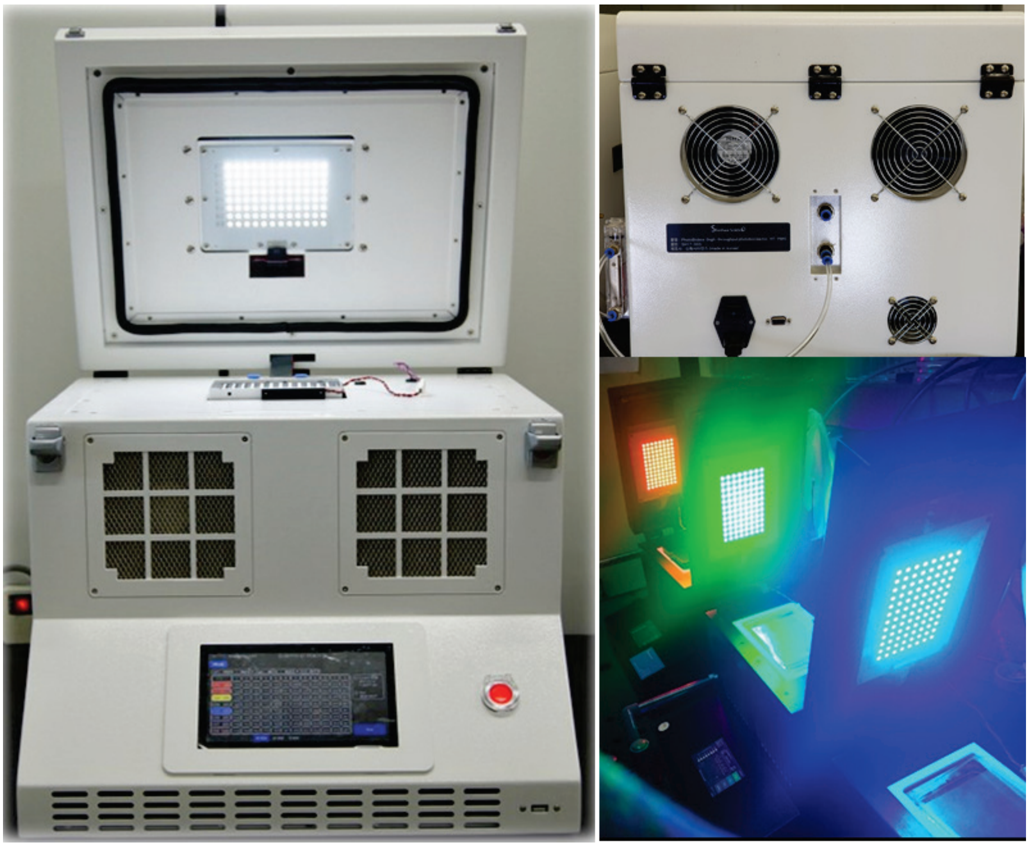

Supplementary data 2. Morphological images, histograms, and scatter plots of each algal strain obtained by FlowCAM and based on three parameters: diameter (μm, X-axes), 10^4 particle per mL (Y-axis of histograms), and aspect ratio (Y-axis of scatter plots). Below, the View Sample window shows the microalgal images in the marked gate (red). **a**) total gate, **b**) *Parachlorella* sp., **c**) *Chlorella* sp., **d**, **e**) *Scenedesmus* sp. and **f**) cell debris.

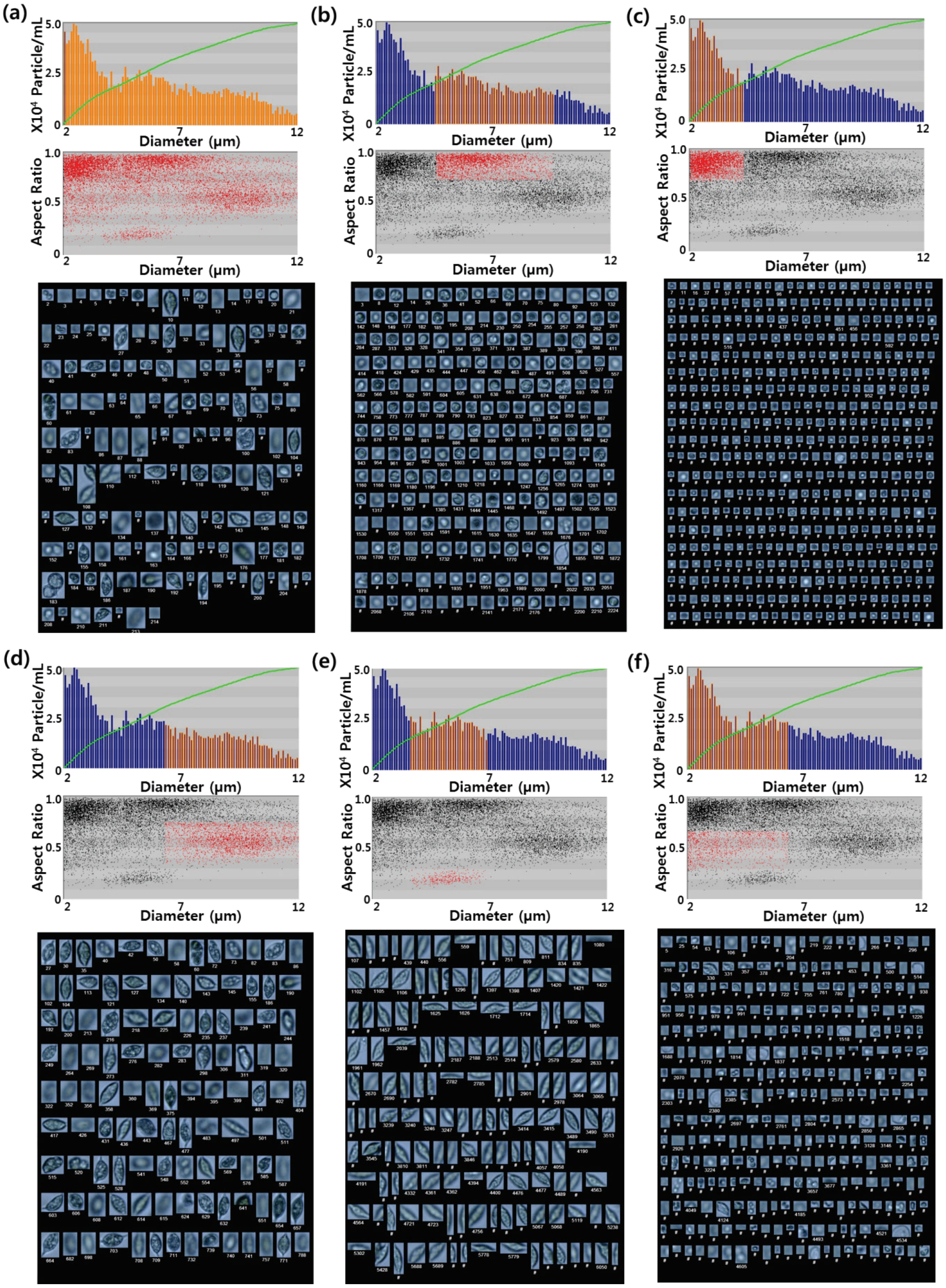

Supplementary data 3. Heat map images representing daily changes (A; 1 day, B; 2 days, and C: 3 days) in the absorbance values (at 680 nm) of an algal culture composed of three algal species including *Chlorella* sp., *Scenedesmus* sp., and *Parachlorella* sp. under air-flow conditions in the PhotoBiobox.

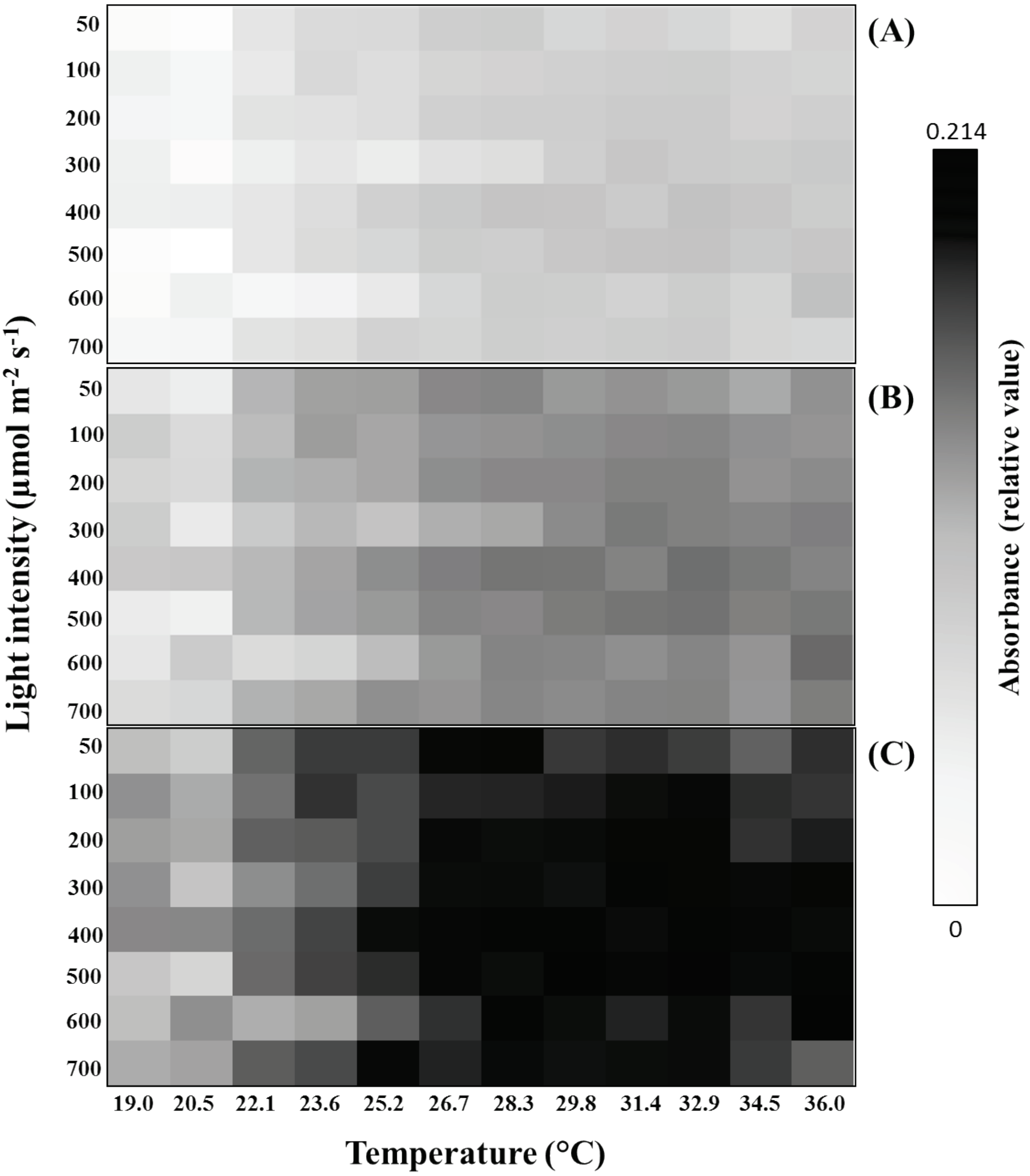

Supplementary data 4. Heat map images representing daily changes (A; 1 day, B; 2 days, and C: 3 days) in the absorbance values (at 680 nm) of an algal culture composed of three algal species including *Chlorella* sp., *Scenedesmus* sp., and *Parachlorella* sp. under 5% CO_2-flow conditions in the PhotoBiobox.

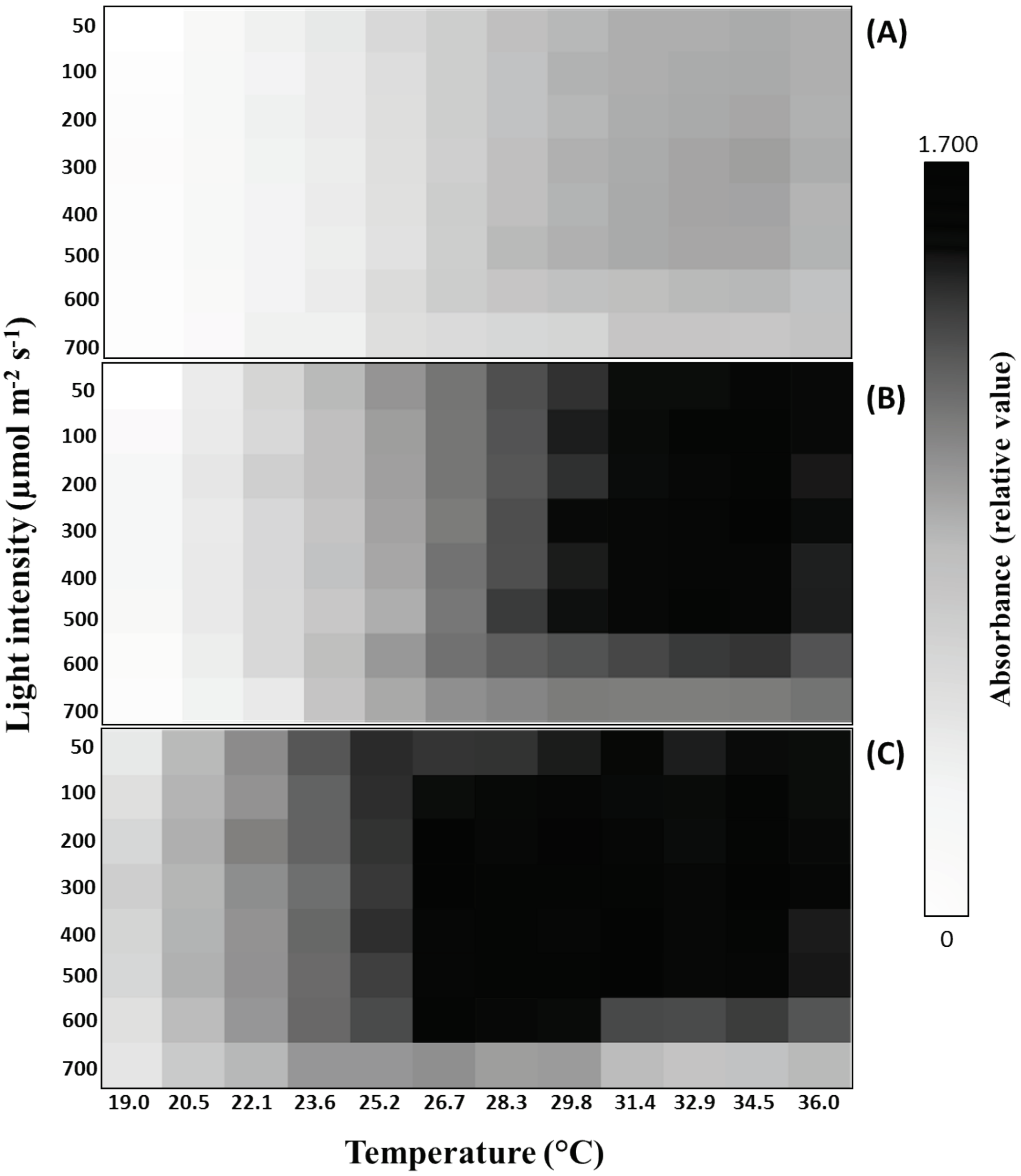

Supplementary data 5. Intraclass correlation coefficients was calculated based on the Cronbach's alpha and ICC model using SPSS version 18.0 for triplicate experiments (for Fig. 3). ($\alpha \geq 0.9$, excellent; $0.9 > \alpha \geq 0.8$, good; $0.8 > \alpha \geq 0.7$, acceptable; $0.7 > \alpha \geq 0.6$, questionable; $0.6 > \alpha \geq 0.5$, poor; $0.5 > \alpha$, unacceptable).

Intraclass Correlation Coefficient

	Intraclass Correlation[a]	95% Confidence Interval		F Test with True Value 0			
		Lower Bound	Upper Bound	Value	df1	df2	Sig
Single Measures	.945[b]	.924	.961	52.783	95	190	.000
Average Measures	.981	.973	.987	52.783	95	190	.000

Two-way random effects model where both people effects and measures effects are random.

a. Type C intraclass correlation coefficients using a consistency definition-the between-measure variance is excluded from the denominator variance.

b. The estimator is the same, whether the interaction effect is present or not.

Supplementary data 6. Intraclass correlation coefficients was calculated based on the Cronbach's alpha and ICC model using SPSS version 18.0. At least triplicates for each assay were performed for triplicate experiments (for Fig. 4). ($\alpha \geq 0.9$, excellent; $0.9 > \alpha \geq 0.8$, good; $0.8 > \alpha \geq 0.7$, acceptable; $0.7 > \alpha \geq 0.6$, questionable; $0.6 > \alpha \geq 0.5$, poor; $0.5 > \alpha$, unacceptable).

Intraclass Correlation Coefficient

	Intraclass Correlation[a]	95% Confidence Interval		F Test with True Value 0			
		Lower Bound	Upper Bound	Value	df1	df2	Sig
Single Measures	.990[b]	.986	.993	292.027	95	190	.000
Average Measures	.997	.995	.998	292.027	95	190	.000

Two-way random effects model where both people effects and measures effects are random.

a. Type C intraclass correlation coefficients using a consistency definition-the between-measure variance is excluded from the denominator variance.

b. The estimator is the same, whether the interaction effect is present or not.

Supplementary data 7. Intraclass correlation coefficients was calculated based on the Cronbach's alpha and ICC model using SPSS version 18.0. At least triplicates for each assay were performed for triplicate experiments. (α≥0.9, excellent; 0.9>α≥0.8, good; 0.8>α≥0.7, acceptable; 0.7>α≥0.6, questionable; 0.6>α≥0.5, poor; 0.5> α, unacceptable).

	Intraclass correlation		*P* value
	Single measures	Average measures (Cronbach's α)	
Air condition	0.945	0.981	0.001
High CO2 condition	0.990	0.997	0.001

Supplementary data 8. Change of PCS compositional values of *Parachlorella* sp. (■), *Chlorella* sp. (■), and *Scenedesmus* sp. (■) by temperature and light intensity under air (A, B) and 5% CO2 (B,C) conditions.

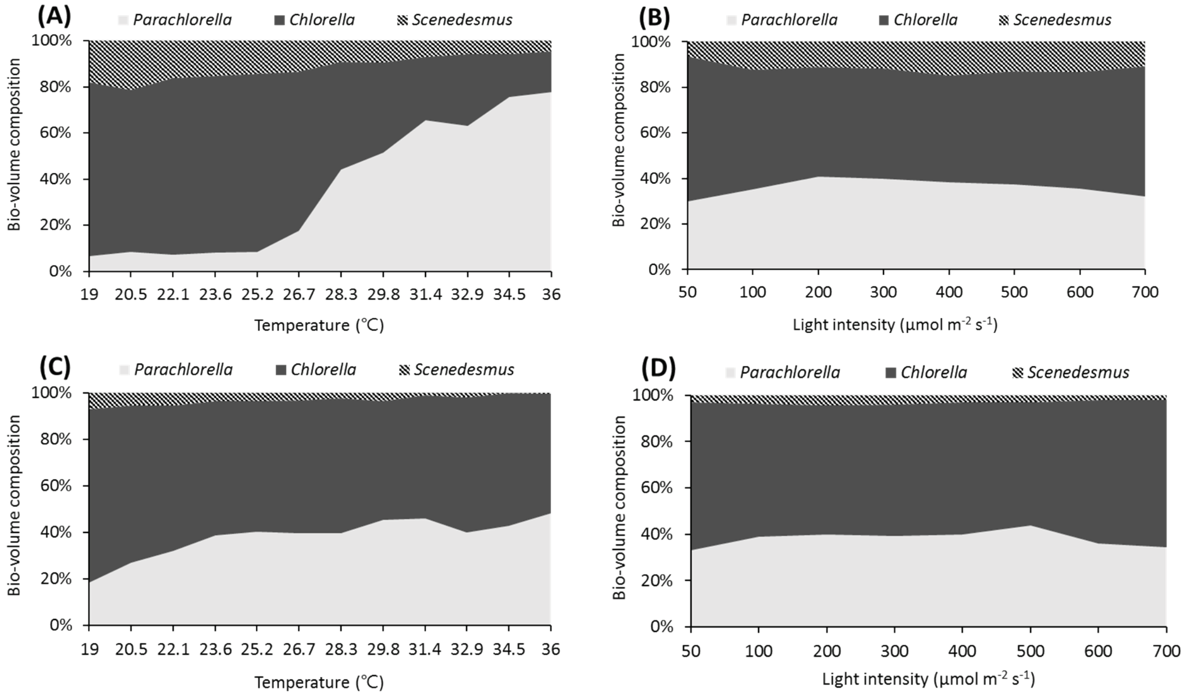

Supplement: Supplementary file 1 [file JMB-30-11-1785-supple.pdf]
